# Frequent Statement and Dereference Elimination for Imperative and Object-Oriented Distributed Programs

**DOI:** 10.1155/2014/839121

**Published:** 2014-04-27

**Authors:** Mohamed A. El-Zawawy

**Affiliations:** ^1^College of Computer and Information Sciences, Al Imam Mohammad Ibn Saud Islamic University (IMSIU), Riyadh 11432, Saudi Arabia; ^2^Department of Mathematics, Faculty of Science, Cairo University, Giza 12613, Egypt

## Abstract

This paper introduces new approaches for the analysis of *frequent statement and dereference elimination* for imperative and object-oriented distributed programs running on parallel machines equipped with hierarchical memories. The paper uses languages whose address spaces are globally partitioned. Distributed programs allow defining data layout and threads writing to and reading from other thread memories. Three type systems (for imperative distributed programs) are the tools of the proposed techniques. The first type system defines for every program point a set of calculated (*ready*) statements and memory accesses. The second type system uses an enriched version of types of the first type system and determines which of the *ready* statements and memory accesses are used later in the program. The third type system uses the information gather so far to eliminate unnecessary statement computations and memory accesses (the analysis of *frequent statement and dereference elimination*). Extensions to these type systems are also presented to cover object-oriented distributed programs. Two advantages of our work over related work are the following. The hierarchical style of concurrent parallel computers is similar to the memory model used in this paper. In our approach, each analysis result is assigned a type derivation (serves as a correctness proof).

## 1. Introduction

Distributed programming is about building a software that has concurrent processes cooperating in achieving some task. For a problem specification, the type, number, and the way of interaction of processes needed to solve the problem are decided beforehand. Then a supercomputer can be computationally simulated by a group of workstations to carry different processes. A group of supercomputers can in turn be combined to provide a computing power greater than that provided by any single machine. This enormous computing power provided by distributed systems is why the distributed programming style [1–3] is quite important and attractive. Among examples of distributed programming languages (DPLs), based on machines having multicore processors and using partitioned-global model, are Unified Parallel C (UPC), Chapel, Titanium which is based on Java, and X10.

Among advantages of object-oriented programming (OOP) is combining other styles such as imperative, functional, and relational programming. Concepts of class, procedure, and inheritance are basics for OOP. These concepts result in dynamic behavior in various implementations of object-oriented programming languages.

Recomputing a nontrivial statement and reaccessing a memory location are waste of time and power if the value of the statement and the content of the location have not been changed. The purpose of* frequent statement and dereference elimination* analysis is to save such wasted power and time. This is an interesting analysis because it involves connecting statement and dereference calculations to program points where the calculated values may be reused. The analysis also requires changing program points at the ends of these connections. Such changes to program points have to be done carefully so that they do not destroy the compositionality. Our approach to treat this analysis is a type system [4, 5] built on a combination of two analyses; one of them builds on the results of the other one.

For different programming languages, in previous work [4, 5], we have proved that the type systems style is certainly an adaptable approach for achieving many static analyses. This paper proves that this style is flexibly useful to the involved and important problem of* frequent statement and dereference elimination* of imperative and object-oriented distributed programs.

This paper introduces new techniques for* frequent statement and dereference elimination* for imperative and object-oriented distributed programs running on hierarchical memories. Simply structured type systems are the main tools of this paper's techniques presented using the languages *while*_*d*_ of Figure 2 and* OODP* of Figure 3. These languages are equipped with basic commands for distributed execution of programs and for pointer manipulations. The single program multiple data (*SPMD*) model is the execution archetypal used in this paper. On different data of different machines this archetypal runs the same program. The analysis of* frequent statement and dereference elimination* for distributed programs is achieved in three steps each of which is done using a type system. The first of these steps achieves* ready statement and memory access* analysis. The second step deals with* semiexpectation* analysis and builds on the type system of the first step. The third type system takes care of the analysis of* frequent statement and dereference elimination* and is built on the type system of the second step. The paper also illustrates how these type systems can be generalized to cover object-oriented distributed languages.

This paper is an extended and revised version of [6], which treats imperative distributed programs. The work of [6] was generalized in Section 5 of the current paper to cover object-oriented distributed programs. The soundness theorems of the current paper are stated using memory model and operational semantics in the appendix of [6].

**Figure 1:**
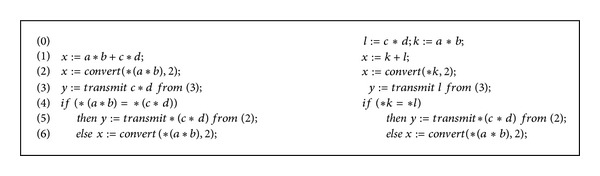
Motivating example.

*Motivation.* The left-hand-side of Figure 1 presents a motivating example of our work. We note that lines 4 and 6 dereference *a*∗*b* which has already been dereferenced in line 2 with no changes to values of *a* and *b* in the path from 2 to 6. This is a waste of computational power and time (accessing a secondary storage). One objective of the research in this paper is to avoid such waste by transforming the program into that in the right-hand-side of the algorithm. This is not all; we need to do that in a way that provides a correctness proof for each transformation. We adopt a style (type systems) that provides these proofs (type derivations).

*Contributions*. Contributions of this paper are new techniques, in the form of type systems, for achieving the following analyses for imperative and object-oriented distributed programs.

1. The analysis of* ready statement and memory access. *
2. The analysis of* semiexpectation*.
3. The analysis of* frequent statement and dereference elimination.*

*Organization*. The rest of the paper is organized as follows.  Section 2 presents the type system achieving the analysis of* ready statement and memory access* for imperative distributed programs. The analysis of* semiexpectation* as an enrichment of the type system presented in Section 2 is outlined in Section 3. The main type system carrying the analysis of* frequent statement and dereference elimination* is contained in Section 4. Type systems of Sections 2, 3, and 4 are generalized in Section 5 to cover object-oriented distributed programs. Related and future works are discussed in Section 6.

## 2. Ready Statement and Memory Access Analysis of *while*_*d*_

If the value of a statement and the content of a memory location have not been changed, then the compiler should not recompute the statement or reaccess the location. The purpose of* frequent statement and dereference elimination* is to save the wasted power and time involved in these repeated computations. This is not a trivial task; compared to other program analyses, it is a bit complex. This task is done in stages. The first stage is to analyze the given program to recognize* ready* statements and memory locations.

The analysis of* ready* statements and memory locations calculates for every program point the set of statements and memory locations that are* ready* at that point in the sense of Definition 1. This section presents a type system (*ready* type system) to achieve this analysis for imperative distributed programs.

**Definition 1**

(1) At a program point *pt*, a statement *S* is* ready* if each computational path to *pt*

- contains an evaluation of *S* at some point (say *pt*′) and
- does not modify *S* (changing value of any of *S*'s variables) between *pt*′ and *pt*.

(2) At a program point *pt*, a memory location *l* is* ready* if each computational path to *pt*

- reads *l* at some point (say *pt*′) and
- does not modify content of *l* between *pt*′ and *pt*.

The* ready* analysis is a forward analysis that takes as an input a set of statements and memory locations (the* ready* set of the first program point). It is sensible to let this set be the empty set. The set of types of our* ready* type system has the form: *points*-*to*-*types* × *P*(*Stmt* ^+^ ∪ *gAddrs*), where

**Figure 2:**
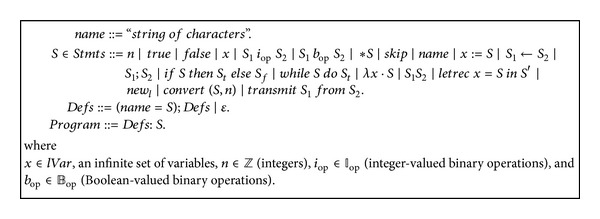
The programming language *while*_*d*_.

1. *Stmt* ^+^ is the set of nontrivial statements (Figure 2),
2. *gAddrs* is the set of global addresses. This set is defined precisely in the appendix of [6], and
3. *points-to-types* is a set of points-to-types (typically have the form of maps from the union of variables and global addresses to the power set of global addresses [4, 7]).

The subtyping relation has the form ≤_*p*_ ×⊇, where ≤_*p*_ is the order relation on the points-to-types and ⊇ is the order relation on *P*(*Stmt* ^+^ ∪ *gAddrs*). A state on an execution path is of type *rs* ∈ *P*(*Stmt* ^+^ ∪ *gAddrs*) if all elements of *rs* are* ready* at this state according to Definition 1. Judgments of the* ready* type system have the form *S* : (*p*, *rs*)→_*m*_(*A*′, *p*′, *rs*′). The symbols *p* and *p*′ denote the points-to-types of the before and after states of executing *S*. The set *A*′ denotes the set of addresses that *S* may evaluate. We assume that all such pointer information is given along with the statement *S*. Techniques like [4, 7] are available to compute the pointer information. For a given statement along with pointer information and a* ready* pretype* rs*, we present a type system to calculate a post* ready*-type *rs*′ such that *S* : (*p*, *rs*)→_*m*_(*A*′, *p*′, *rs*′). The type derivation of this typing process is a proof for the correctness of the* ready* information. The meaning of the judgment is that if elements of *rs* are* ready* before executing *S*, then elements of *rs*′ are* ready* after executing *S*.

The inference rules of the* ready* type system are presented in Algorithm 1. Comments on the inference rules are in order. We note that numbers, variables, and the allocating statement (*new*) do not affect the* ready* pretype. In line with semantic rules (*i*_*op*_^*r*^) and (*b*_*op*_^*r*^) [6], nontrivial arithmetic and Boolean statements and their nontrivial substatements are made* ready*. The direct assignment rule (∶=^*r*^) expresses that after executing the assignment the substatements of r.h.s. become* ready* and that all statements involving *x* become* unready* as the value of *x* may become different. The rule (∗^*r*^) reflects the fact that the statement ∗*S* becomes* ready* after executing the dereference. Moreover if *S* evaluates a single address according to the underlying pointer analysis, then this address becomes* ready* as well. However if *S* evaluates a large set of addresses (more than one), then we are not sure which of these addresses is the concerned one and hence cannot conclude any readiness information about addresses. The rule (←^*r*^) adds the substatements of *S*_1_ and *S*_2_ to the* ready* pretype. Since the content of address referenced by *S*_1_ is possibly changed after executing the statement, all statements involving dereferencing this address are removed from the set of* ready* items. Remaining rules are self-explanatory. The Boolean statements *true* and *false* have inference rules similar to that of *n*.

All in all, the information provided by type derivations obtained using this and the following type system is classified into two sorts. The first sort is about knowing the program point at which a particular statement becomes* ready*. The second sort of information is about the program point at which a precomputed value of a* ready* statement can be replaced with the statement.

Now we recall the assumption that our distributed system consists of |*M*| machines. For a given statement *S* and a given machine *m*, the type system of Algorithm 1 calculates for each program point of *S*, the set of* ready* items. The following rule can be used to combine the information calculated for each machine to get new* ready* information for each program point. The new* ready* information is valid on any of the |*M*| machines.

Consider

(1) ∀m∈M·S:(sup⁡⁡{p,pj ∣ j≠i},sup⁡⁡{rs,rsj ∣ j≠i})⟶m(Am,pm,rsm)S:(p,rs)⟶M(∪iAi,sup⁡⁡{p1,…,pn},sup⁡⁡{rs1,…,rsn})(main-rs).

The rule (*main-rs*) supposes a suitable notion for the join of pointer types. The soundness of the* ready* type system is stated as follows.

**Theorem 2**

Suppose that (*S*, *δ*)→(*V*, *δ*′), *S* : (*p*, *rs*)→(*A*′, *p*′, *rs*′), and the items of *rs* are* ready* at the point corresponding to *δ* on the execution path. Then the items of *rs*′ are* ready* at the point corresponding to *δ*′ on the execution path.

## 3. *Semiexpectation* Analysis of *while*_*d*_

The aim of frequent statement elimination is to introduce new variables to accommodate values of frequent statements and reusing these values rather than recomputing the statements. Analogously, the aim of frequent dereferences elimination is to introduce new variables to accommodate values of frequent dereferences and reusing these values rather than reaccessing the memory. The information gathered so far by the* ready* type system introduced in the previous section is not enough to achieve frequent statements and dereferences elimination. We need to enrich the* ready* information, assigned to each program point, with new information called* semiexpectable* information.

**Definition 3**

(1) At a program point *p*, a statement *S* is* semiexpectable* if there is a computational path from *p* that

- contains an evaluation of *S* at some point (say *p*′), where *S* is ready at *p*′, and
- does not evaluate *S* between *p*′ and *p*.

(2) At a program point *p*, a memory location *l* is* semiexpectable* if each computational path to *p*

- reads *l* at some point (say *p*′) where *l* is ready at *p*′, and
- does not read *l* between *p*′ and *p*.

The* semiexpectation* analysis is a backward analysis that takes as an input a set of statements and memory locations (the* semiexpectable* set of the last program point). It is sensible to let this set be the empty set. The following example gives an intuition for the previous definition:

(2) $$\begin{array}{c} \text{if}\left(\cdots \right),\text{then}\;a∶=y+t\;\text{else}\;b∶=*r;\;c∶=\frac{\left(y+t\right)}{*r}. \end{array}$$

Neither the statement *y* + *t* nor the statement ∗*r* is* ready* after the if statement because they are not computed in all branches. Hence it is not true to replace these statements with variables towards optimizing the last statement of the example. The job of the type system presented in this section is to provide us with this sort of information. More precisely, as the statements *y* + *t* and ∗*r* are not* ready* after the if statement, the second statement of the example does not make them* semiexpectable*.

The* semiexpectation* analysis assigns for each program point the set of items that are* semiexpectable*. The analysis is based on the* readiness* analysis and is backward. The set of types of the* semiexpectation* type system has the form: *points*-*to*-*types* × *P*(*Stmt* ^+^ ∪ *gAddrs*) × *P*(*Stmt* ^+^ ∪  *gAddrs*). The subtyping relation has the form ≤_*p*_ × ⊇×⊇. A state on an execution path is of type *se* ∈ *P*(*Stmt* ^+^ ∪ *gAddrs*) if all elements of *se* are* semiexpectable* according to Definition 3. Judgments of the* semiexpectation* type system have the form *S* : (*p*, *rs*, *se*)→_*m*_(*A*′, *p*′, *rs*′, *se*′). For a given statement along with pointer information, readiness information, and a* semiexpectation* type *se*′, we present a type system to calculate a pre-*semiexpectable*-type *se* such that *S* : (*p*, *rs*, *se*)→_*m*_(*A*′, *p*′, *rs*′, *se*′). The type derivation of this typing process is proof for the correctness of the* semiexpectable* information. The meaning of the judgment is that if elements of *se*′ are* semiexpectable* after executing *S*, then elements of *se* must have been* semiexpectable* before executing *S*.

The inference rules of the* semiexpectation* type system are shown in Algorithm 2. Some comments on the inference rules are in order. In the rule (*i*_*op*_^*e*^), given the posttype *se*′, we calculate the pretype *se*′′ for the statement *S*_2_. Then the resulting pretype is used as a posttype for the statement *S*_1_ to calculate the pretype *se*. In line with Definition 3, the arithmetic statement *S*_1_ *i*_*op*_ *S*_2_ is added to *se* only if it belongs to *rs*. Similar explanations illustrate the rule (∗^*e*^). The remaining rules mimic the rules of the* ready* type system.

Now we recall the assumption that our distributed system consists of |*M*| machines. For a given statement *S* and a given machine *m*, the type system given above calculates for each program point of *S* the set of* semiexpectable* items. Now the following rule can be used to combine the information calculated for each machine to get new* semiexpectable* information for each program point. The new* semiexpectable* information is valid on any of the |*M*| machines.

Consider

(3) ∀m∈M·S:(sup⁡{p,pj ∣ j≠i},sup⁡⁡{rs,rsj ∣ j≠i},sem)⟶m(Am,pm,rsm,inf⁡⁡{se′,sej ∣ j≠i})S:(p,rs,inf⁡⁡{se1,…,se|M|})⟶M(∪iAi,sup⁡⁡{p1,…,p|M|},sup⁡⁡{rs1,…,rs|M|},se′)(main-rs).

The difference in the way that this rule treats the* semiexpectable* information and the way* ready* information is treated is explained by the fact that the* ready* analysis is forward while the* semiexpectation* analysis is backward.

It is not hard to prove the soundness of the above type system.

**Theorem 4**

Suppose that (*S*, *δ*)→(*V*, *δ*′), *S* : (*p*, *rs*, *se*)→(*A*′, *p*′, *rs*′, *se*′) and the items of *se*′ are* semiexpectable* at the point corresponding to *δ*′ on the execution path. Then the items of *se* are* semiexpectable* at the point corresponding to *δ* on the execution path.

## 4. Frequent Statement and Dereference Elimination of *while*_*d*_

This section presents a type system that is an enrichment of the type system presented in the previous section. The type system of this section achieves the* frequent statement and dereference elimination*. The type system uses a function *sn* : *S* ^+^ → *Stmt*-*names* that assigns each nontrivial statement a name. These names are meant to carry values of frequent statements and dereferences. The judgments of our type system have the form *S* : (*p*, *rs*, *se*)→_*m*_(*A*′, *p*′, *rs*′, *se*′)⇛(*ns*, *S*′). The type information (*p*, *rs*, *se*) and (*A*′, *p*′, *rs*′, *se*′) were calculated by the previous type system. *S*′ is the optimization of *S* and *ns* is a sequence of assignments that links optimized statements with the names of their unoptimized versions.

Algorithms 3 and 4 present inference rules for the* frequent statements and dereferences elimination*. We note the following on the inference rules. A big deal of optimization is achieved by the three rules for ∗*S*. These rules are (∗_1_^*f*^), (∗_2_^*f*^), and (∗_3_^*f*^). The rule (∗_1_^*f*^) takes care of the case where ∗*S* is* ready* and is replaceable by its name under the function *sn*. The rule (∗_2_^*f*^) treats the case where ∗*S* is* semiexpectable* and is not* ready* before calculating the statement. In this case, a statement name of ∗*S* is used. The rule (∗_3_^*f*^) considers the case where ∗*S* is neither* semiexpectable* at the program point after execution nor* ready* before calculating the statement. In this case, the statement ∗*S* does not get changed. Similarly, the three rules (*i*_*op*(1)_^*f*^), (*i*_*op*(2)_^*f*^), and (*i*_*op*(3)_^*f*^) treat different cases for arithmetic statements. The Boolean statements are treated with rules quite similar to that of arithmetic statements. The rule (*whl* ^*f*^) reuses frequent substatements of the guard. This is done via adding *ns* in the positions clarified in the rule. Remaining rules of system are self-explanatory.

For expressing the soundness, we introduce the following definition.

**Definition 5**

Suppose that *δ* is a state defined on the set of locations,* Loc* ([6, Definition 4]). Suppose also that *δ*_∗_ is a state defined on *Loc* ∪ *Stmt*-*names*. The expression *δ* ≡_*se*_ *δ*_∗_ denotes the fact that *δ* and *δ*_∗_ are equivalent with respect to the* semiexpectation* type* se*. More precisely *δ* ≡_*se*_ *δ*_∗_ if and only if

1. for  all  *j* ∈ *Loc*.  *δ*(*j*) = *δ*_∗_(*j*), and
2. for  all  *S* ∈ *se*.  (*S*, *δ*)⇝_*m*_(*v*, *δ*′)⇒*δ*_∗_(*sn*(*S*)) = *v*.

The soundness of* frequent statements and dereferences elimination* means that the original and optimized programs are equivalent in the following sense:

- the states of the two programs coincide on the* Loc*, and
- if a statement is both* ready* and* semiexpectable*, then its semantics in the original-program state equals the value of its corresponding name in optimized-program state.

This gives an intuition to the previous definition. The following soundness theorem is proved by a structure induction.

**Theorem 6**

Suppose that *S* : (*p*, *rs*, *se*)→_*m*_(*A*′, *p*′, *rs*′, *se*′)⇛(*ns*, *S*′) and *δ* ≡_*se*_ *δ*_∗_. Then

- (*S*, *δ*)⇝_*m*_(*v*, *δ*′)⇒∃*δ*_∗_′.  *δ*′≡_*se*′_ *δ*_∗_′ *and* (*S*′, *δ*_∗_)⇝_*m*_(*v*, *δ*_∗_′);
- (*S*′, *δ*_∗_)⇝_*m*_(*v*, *δ*_∗_′)⇒∃*δ*′.  *δ*′≡_*se*′_ *δ*_∗_′ *and* (*S*, *δ*)⇝_*m*_(*v*, *δ*′).

## 5. Frequent Statement and Dereference Elimination of* OODP* Programs

**Figure 3:**
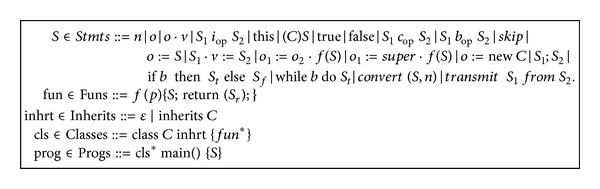
The programming language* OODP*.

This section generalizes the type systems of previous sections to cover object-oriented distributed programs. Hence, a new model for object-oriented distributed programs and necessary changes to proposed type systems for the analysis of* frequent statement and dereference elimination* are presented in this section. Object-oriented concepts such as subtyping and inheritance are included in the model language (dubbed* OODP*) whose syntax is shown in Figure 3.

**Figure 4:**
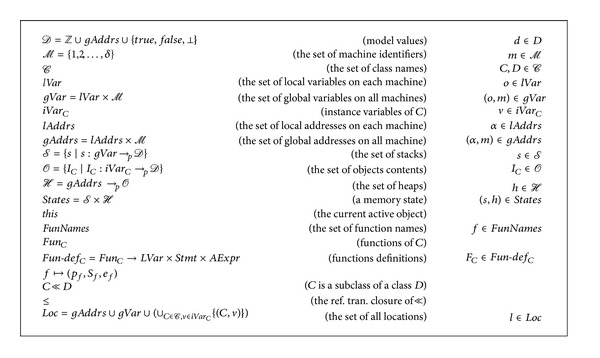
Semantic spaces and naming conventions.

In line with OOP concepts, local variables are contained in functions and live while their functions are live. While parameters of function are represented using local variables, a class's internal state is contained in its instance variables. A class is a container for a set of function definitions. Each function *f* has parameter *p*_*f*_, a main statement *S*_*f*_, and a statement *S*_*f*_ representing value returned by the function. Hence an* OODP* program is a set of classes followed by a “main” function.  Figure 4 presents semantic spaces and naming conventions used in the rest of the paper.

As shown in the previous sections, the analysis of* frequent statement and dereference elimination *for imperative distributed programs is achieved in three steps. In the following, we show necessary changes to the three type systems presented so far to cover object-oriented distributed programs.

For each program point,* ready* statements and memory locations (Definition 1) are computed by the analysis of* ready* statements and memory locations. Adding rules of Algorithm 5 to that of Algorithm 1 results in a type system that calculates this analysis for object-oriented distributed programs of Figure 3. Using semantics notions of Figure 4, Definitions 1, 3, and 5 are applicable and convenient for the analyses in this section for the language* OODP*.

Comments on the inference rules are in order. The rules of Algorithm 5 suppose the existence of a* class* analysis that calculates the set of classes that a statement may reference. The judgments of the proposed analysis have the form *S* : *p* → *Cs*. The intuition of such judgments is that the pointer information are used to calculate the set *Cs*. In the rule (∶=_*S*·*v*_^*r*^),* ready* substatements of *S*_1_ and *S*_2_ are added to *rs* to produce *rs*′. Then for any class *C* that *S*_1_ may reference, statements involving *C* · *v* are removed from *rs*′. In the rule (∶=_*o*·*f*_^*r*^), *Cs* includes classes that *o*_2_ may reference. For all functions named *f* in classes of *Cs*, the body and return statements are enumerated in the set {*S*_1_,…, *S*_*m*_}.* Ready* substatements of these statements are added to *rs* to produce *rs*_*m*+1_. Then all statements involving *o*_1_ are removed from *rs*_*m*+1_.

Using semantics notations of Figure 4, soundness of the type system of Algorithm 5 is stated as follows.

**Theorem 7**

Suppose that (*S*, *s*, *h*)→(*V*, *s*′, *h*′), *S* : (*p*, *rs*)→(*A*′, *p*′, *rs*′) and the items of *rs* are* ready* at the point corresponding to (*s*, *h*) on the execution path. Then the items of *rs*′ are* ready* at the point corresponding to (*s*′, *h*′) on the execution path.

The goals of main analysis of this section for* OODP* are as follows.

Introducing new variables to maintain values of frequent statements and dereferences and then reusing these values instead of recomputing the statements and reaccessing the memory.

To achieve this goal the* ready* information needs to be enriched with information of* semiexpectable*.

Adding rules of Algorithm 6 to that of Algorithm 2 results in a type system that calculates the analysis of* semiexpectation* for object-oriented distributed programs of Figure 3. Some comments on the inference rules of Algorithm 6 are in order. In the rule (∶=_*S*·*v*_^*e*^), starting with the posttype *se*′, the pretype *se*′′ is calculated for the statement *S*_2_. Then *se*′ is used as a posttype for *S*_1_ to get the main pretype *se*. Similarly to (∶=_*o*·*f*_^*r*^), the rule (∶=_*o*·*f*_^*e*^) enumerates body and return statements of convenient functions. Then sequentially *se* is calculated starting from *se*′. The remaining rules mimic the rules of the* ready* type system.

Using semantics notations of Figure 4, soundness of the type system of Algorithm 6 is stated as follows.

**Theorem 8**

Suppose that (*S*, *s*, *h*)→(*V*, *s*′, *h*′), *S* : (*p*, *rs*, *se*)→(*A*′, *p*′, *rs*′, *se*′), and the items of *se*′ are* semiexpectable* at the point corresponding to (*s*′, *h*′) on the execution path. Then the items of *se* are* semiexpectable* at the point corresponding to (*s*, *h*) on the execution path.

Adding rules of Algorithm 7 to that of Algorithm 3 results in the main type system achieving the analysis of* frequent statement and dereference elimination* for object-oriented distributed programs of Figure 3. We note the following on the inference rules. Optimization is based on rules for (*C*)*S*; ((*C*)*S*_1_^*f*^), ((*C*)*S*_2_^*f*^), and ((*C*)*S*_3_^*f*^). The case that (*C*)*S* is* ready* and is replaceable by its name under the function *sn* is treated by ((*C*)*S*_1_^*f*^). The case (*C*)*S* is* semiexpectable* but not* ready* before calculating the statement is treated by ((*C*)*S*_2_^*f*^). The rule ((*C*)*S*_3_^*f*^) takes care of the case, where (*C*)*S* is neither* ready* before the calculation nor* semiexpectable* after execution.

The following definition generalizes Definition 5 and is necessary to express soundness.

**Definition 9**

Suppose that (*s*, *h*) is a state defined on the set of locations* Loc* ([6, Definition 4]). Suppose also that (*s*_∗_, *h*_∗_) is a state defined on *Loc* ∪ *Stmt*-*names*. The expression (*s*, *h*) ≡_*se*_(*s*_∗_, *h*_∗_) denotes the fact that (*s*, *h*) and (*s*_∗_, *h*_∗_) are equivalent with respect to the* semiexpectation* type* se*. More precisely (*s*, *h*) ≡_*se*_(*s*_∗_, *h*_∗_) if and only if

1. for all  *l* ∈ *Loc*.  *s*(*l*) = *s*_∗_(*l*), and
2. for all *S* ∈ *se*.  (*S*, *s*, *h*)→_*m*_(*v*, *s*′, *h*′)⇒*s*_∗_(*sn*(*S*)) = *v*.

Using semantics notations of Figure 4, soundness of the type system of Algorithm 7 is stated as follows.

**Theorem 10**

Suppose that *S* : (*p*, *rs*, *se*)→_*m*_(*A*′, *p*′, *rs*′, *se*′)⇛(*ns*, *S*′) and (*s*, *h*) ≡_*se*_(*s*_∗_, *h*_∗_). Then

- (*S*, *s*, *h*)→_*m*_(*v*, *s*′, *h*′)⇒∃(*s*_∗_′, *h*_∗_′). (*s*′, *h*′) ≡_*se*′_(*s*_∗_′, *h*_∗_′) and (*S*′, *s*_∗_, *h*_∗_)→_*m*_(*v*, *s*_∗_′, *h*_∗_′);
- (*S*′, *s*_∗_, *h*_∗_)→_*m*_(*v*, *s*_∗_′, *h*_∗_′)⇒∃(*s*′, *h*′). (*s*′, *h*′) ≡_*se*′_(*s*_∗_′, *h*_∗_′) and (*S*, *s*, *h*)→_*m*_(*v*, *s*′, *h*′).

## 6. Related Work

The techniques of common subexpression elimination (CSE) [8, 9] are closed to our work. In [10], a type system for CSE of the* while* language is introduced. The work presented in our paper can be realized as a generalization of that presented in [10]. The generality of our work is evident in our language models which are much richer with distributed, pointer, and object-oriented commands. Consequently, the operational semantics that we measure the soundness of our system against are much more involved than that used in [10]. Using new opportunities appearing while scheduling control-intensive designs, the work in [11] introduces a technique that dynamically eliminates CSE. To optimize polynomial expressions (important for applications like domains, computer graphics, and signal processing), the paper [12] generalizes algebraic techniques originally designed for multilevel logic synthesis. The generalization in [12] uses factoring to eliminate common subexpressions of polynomial expressions.

There are many analyses for optimizing object-oriented programs. In [13] evolutionary multiobjective optimization methods are used to present a Class-Based Elitist Genetic Algorithm (CBEGA) for testing OOP. A new method to optimize OOP for field access in concurrent object-oriented programs is presented in [14]. This work utilizes the correctness concept that concurrency control must be used by programmers. A new model concurrency abstraction is presented in [15]. This model has the advantage of separating the specification of the synchronization code from the method bodies.

The association of a correctness proof with each result of the static analysis is important and needed by applications like proof-carrying code and certified code. The work presented in this paper has the advantage over most related work of constructing these proofs. Adding to the value of using type systems, the proofs constructed in our proposed approach have the form of type derivations. The work in [4, 16, 17] presents many examples of other static analyses that are in the form of type systems.

In [18], a technique for flow-insensitive pointer analysis of programs that run on parallel and hierarchical machines and that share memory is introduced. Via a two-level hierarchy, [19, 20] present constraint-based approaches to evaluate locality information and sharing attributes of references. Our language model is a generalization of models presented in [18, 19].

Much research acclivities [18, 21] was devoted to analyze distributed programs. This is motivated by the importance of distributed programming as a main stream of programming today. The examining and capturing of causal and concurrent relationships are among important issues to many distributed systems applications. In [22], an analysis that examines the source code of each process constructs an inclusive graph, POG, of the possible behaviors of systems. Data racing bugs [23] can be a side effect of the parallel access of cores of a multicore process to a physically distributed memory. In [23] a technique, called DRARS, is proposed for avoidance and replay of this data race. Parallel programs on DSM or multicore systems can be debugged using DRARS. The classical problems of satisfiability decidability and algorithmic decidability are approached in [24] on the distributed-programs model of message sending. In this work, distributed programs are represented by communicating via buffers.

## 7. Conclusion

This paper introduces new techniques for the analysis of* frequent statement and dereference elimination* for imperative and object-oriented distributed programs running on parallel machines equipped with hierarchical memories. Type systems are the tools of the techniques presented in this paper. The first sort of proposed type systems defines for program points of a distributed program sets of calculated (*ready*) statements and* memory accesses*. The second sort determines which of the* ready* statements and* memory accesses* are used later in the program. The final sort eliminates unnecessary statement computations and* memory accesses*.

## Figures and Tables

**Algorithm 1:**
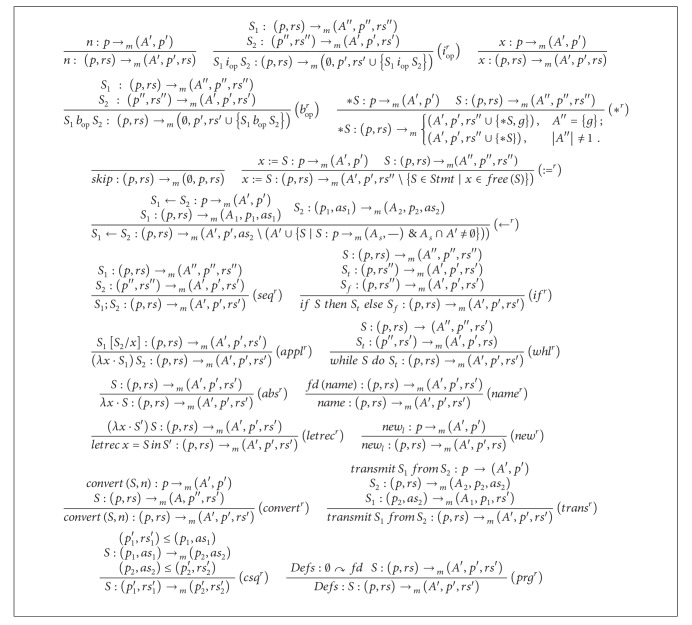
Inference rules of the *ready* type system.

**Algorithm 2:**
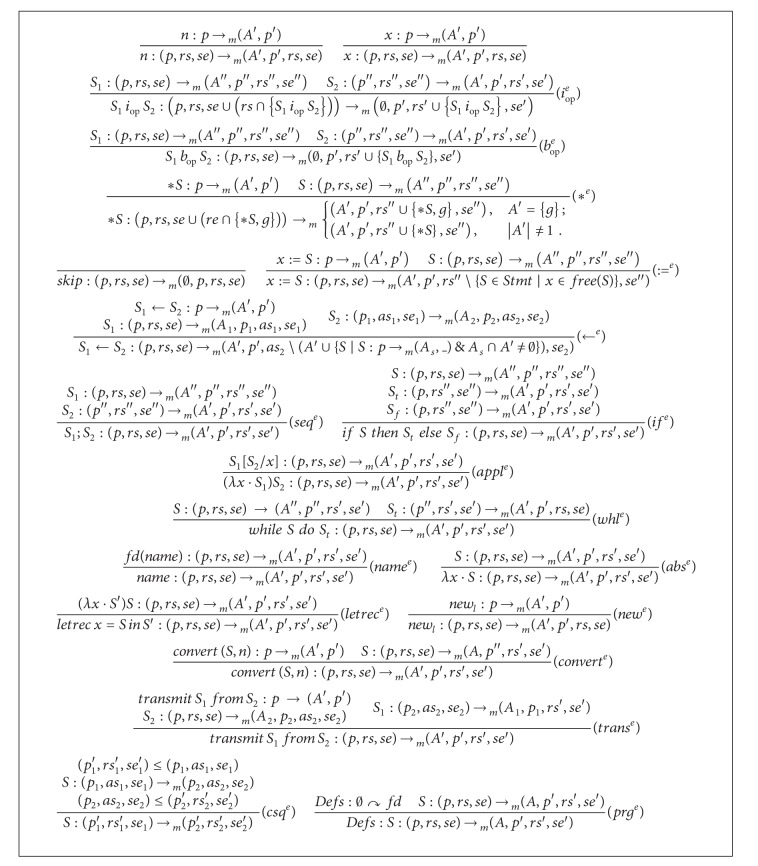
Inference rules of the *semiexpectation *type system.

**Algorithm 3:**
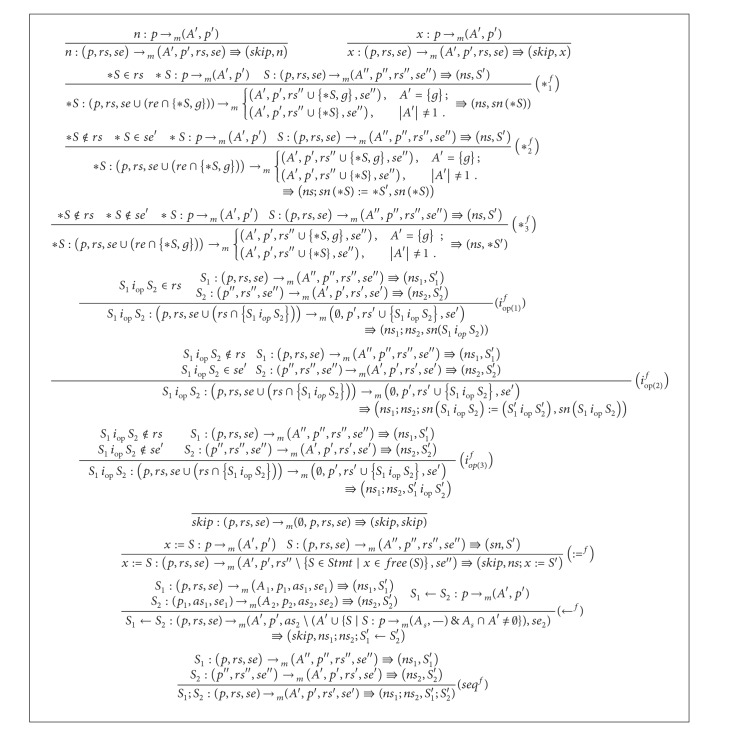
Inference rules for the *frequent statements and dereferences elimination* (1).

**Algorithm 4:**
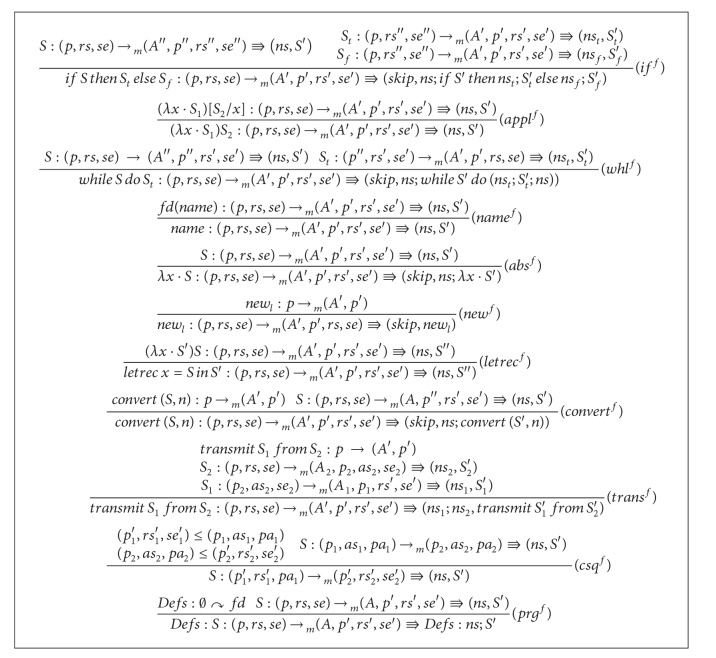
Inference rules for the *frequent statements and dereferences elimination* (2).

**Algorithm 5:**
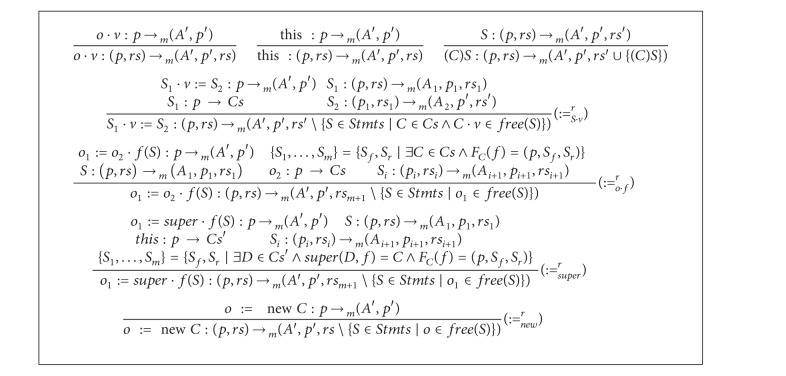
An extension for the *ready* type system to cover *OODP* programs.

**Algorithm 6:**
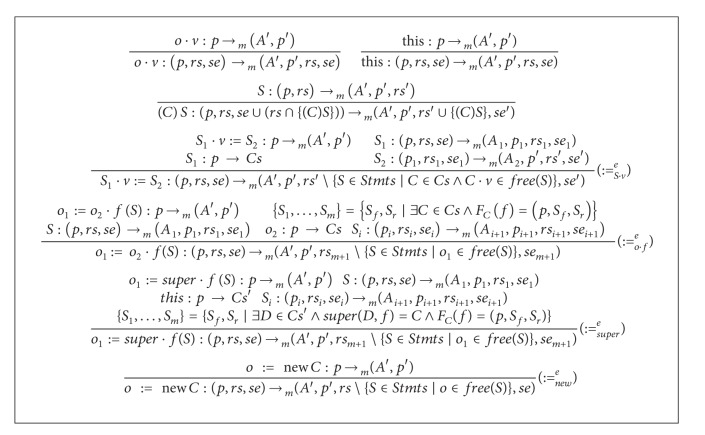
An extension for the *semi-expectation* type system.

**Algorithm 7:**
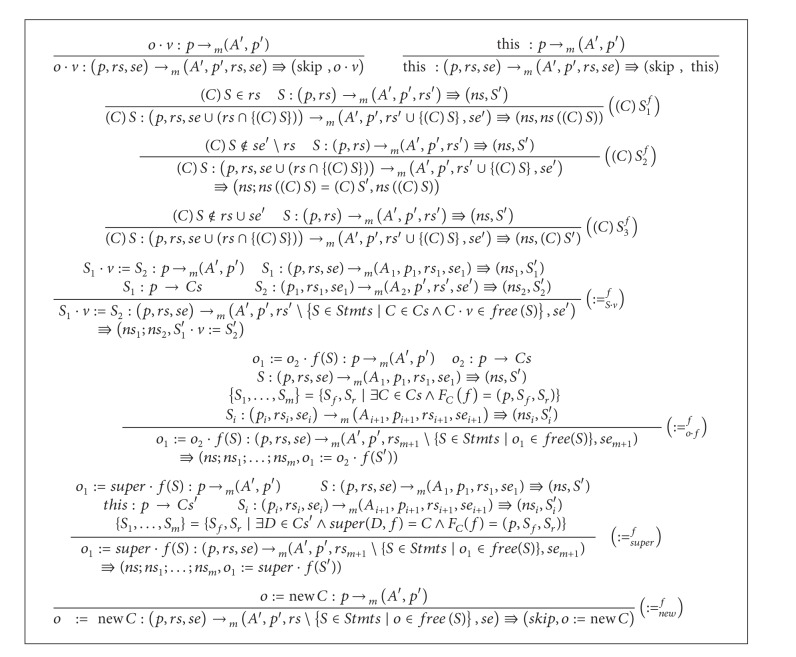
An extension for the *frequent statements and de-references elimination* type system.
